# Factors Associated with Postoperative Pain among Patients after Cardiac Surgery in the Tertiary Care Teaching Hospital of Karachi, Pakistan

**DOI:** 10.1155/2019/9657109

**Published:** 2019-04-01

**Authors:** Sineer Micah, Rubina Barolia, Yasmin Parpio, Santosh Kumar, Hasnat Sharif

**Affiliations:** ^1^Male School of Nursing, Sindh Government Hospital Liaquat Abad, Karachi, Pakistan; ^2^School of Nursing and Midwifery, Aga Khan University, Karachi, Pakistan; ^3^College of Nursing, Jinnah Sindh Medical University (JSMU), Karachi, Pakistan; ^4^Department of Cardiothoracic Surgery, Aga Khan University Hospital, Karachi, Pakistan

## Abstract

**Background:**

Pain is the subjective feeling of an individual, which affects the overall recovery of patients after cardiac surgery. Postoperative pain is the most inadequately managed symptom of cardiac surgery. Subsequently, there are many factors that can either hinder or facilitate pain management, including patients' beliefs, cultural values, physiological features, hospital policies, and healthcare providers' knowledge and beliefs. The purpose of this research was to identify factors associated with postoperative pain and its management, after cardiac surgery, among patients in a tertiary care hospital in Karachi, Pakistan.

**Methods:**

Quantitative correlational study design was employed to attain the study purpose. Data were collected from 136 adult cardiac surgery patients admitted in the Cardiothoracic Surgery (CTS) Department, of tertiary care hospital. A self-developed questionnaire tool was used to gather information from patients. Data was then analyzed on SPSS version 19. Mann-Whitney U, Kruskal Wallis, and Spearman tests were applied to find the associations between the pain levels and of the independent variables.

**Results:**

The mean pain scores of the first, second, and third postoperative days were found to be 2.98, 2.96, and 2.98, respectively. The findings also showed that BMI and the types of surgery were significantly associated with postoperative pain. Patients' beliefs regarding drug dependency, fear of adverse effects, and postoperative physical activities were also associated with pain. Furthermore, the nurses' education level and reluctance in medication administration due to fear of adverse effects were found to be significant too.

**Conclusion:**

The study identified some of the important factors that were associated with postoperative pain. The results suggest the need for the enhancement of patients' education on drug dependency, adverse effects, and physical activity, before cardiac surgery. The nurses should be educated on pain management keeping the patients' culture and other perceptions of pain in mind.

## 1. Introduction

Cardiovascular diseases (CVD) are an area of major health problem worldwide [1]. The burden of CVD is about 80% higher among individuals from low- and middle-income countries (LMICs) and risk of Coronary Artery Disease (CAD) is 3 to 5 times higher in the South Asian population than in other ethnicities [2, 3]. Subsequently, more than 20,000 cardiac surgeries are performed every year in Australia [4] and over 66,000 cardiac surgeries are performed in Turkey yearly [5]. Similarly, in Europe, US, and Canada, 70% of CAD patients undergo CABG and 11% have both CABG and valvular surgery [6, 7].

Pain has been defined as the most important and inadequately managed symptom that affects the overall recovery of cardiac surgery patients [8, 9]. The consequences of unrelieved pain are long-term, and they negatively affect a patient's health from multiple perspectives: they reduce the quality of life, impair sleep, impair physical functions, and increase the economic costs of treatment [10]. There are many patient-related factors that either hinder or facilitate pain management after cardiac surgery, such as demographics, knowledge, beliefs, psychosocial factors, nature of the surgery, and lack of communication with healthcare providers [11, 12]. Evidence suggests that culture is another significant determinant of patient's reaction to pain and their response to managing this pain [6].

The pharmacological management of pain is carried out through the combination of different medications, such as opioids and nonopioids. Furthermore, there are many nonpharmacological methods which are used for the management of postoperative pain such as deep breathing, massaging, relaxing, and music therapy. However, despite using several pharmacological and nonpharmacological interventions, it has been identified that pain is inadequately managed after cardiac surgery [6, 13, 14].

The prevalence of sternal incision pain from cardiac surgery has been reported to be around 20 to 50% during the first and second weeks following surgery and hospital discharge [15, 16]. Therefore, it is important to identify the factors associated with postoperative pain after cardiac surgery, as these factors result in psychological and physiological consequences, such as atelectasis and hemodynamic instability, and depression [17]. In addition, pain management is also related to a patient's recovery after surgery because patients are required to perform certain postoperative activities, such as deep breathing, incentive spirometry, and out-of-bed ambulation, to foster fast recovery and early hospital discharge [18]. Therefore, it is imperative to assess the factors associated with postoperative pain management in the clinical settings to help clinicians to improve compliance with these postoperative activities. The present study aimed to identify factors associated with postoperative pain and its management, after cardiac surgery, among patients in a tertiary care hospital of Karachi, Pakistan.

## 2. Methodology

### 2.1. Study Design and Setting

Quantitative correlational study design was used to assess the factors associated with postoperative pain among patients after cardiac surgery. The study was conducted at Cardiothoracic Surgery (CTS) department of the Aga Khan University Hospital (AKUH), Karachi, Pakistan. The AKUH is a tertiary care hospital with more than 600 inpatient beds.

### 2.2. Study Population

The study was conducted among patients admitted in the department of CTS from April to June 2016. The target population of this study included adult patients who had undergone any type of cardiac surgery, such as Coronary Artery Bypass Grafting (CABG), Valve Replacement/Repair Surgeries, and Congenital Heart Surgeries. Patients who followed the normal pathway of cardiac surgery were included in the study. The cardiac surgery pathway is the AKUH document used for the development of a clinical management plan, shows the fast track recovery goals for patients, and ensures the provision of ideal and systemic nursing actions to attain these goals with maximum efficiency. The format of a pathway provides the framework for the healthcare providers to focus on the step-by-step important components of care to be maintained on each postoperative day.

### 2.3. Eligibility Criteria

Patients who were 18 years or older, followed the cardiac clinical normal pathway, and were ready for cardiac rehabilitation (3rd-4th postoperative day) were included in the study. Those patients who were unable to comprehend Urdu or English language and had altered conscious level and Glasgow Coma Scale (GCS) of less than 15 were excluded. In addition, those patients who were receiving intravenous opioids on the 3rd-4th postoperative day and had severe respiratory distress and hemodynamic instability were excluded from the study.

### 2.4. Sample Size

The sample size was computed using the Open Epi Software version 3.03. In order to determine the factors associated with postoperative pain and its management, the variable of physical activity was considered. Keeping the confidence level at 95%, power at 80%, odds ratio (OR) as 0.25, and the frequency of pain among patients not rested (nonexposed) was taken into consideration. Finally, the sample size that emerged was 202 participants. This sample size was further inflated by 10% to wipe out the effects of errors, nonresponse, and missing data. Hence, the final sample size was taken as 223 participants.

### 2.5. Data Collection and Quality Assurance of the Tool

Data was collected by using a structured questionnaire; the questionnaire was self-developed and was divided into two sections. The demographic and clinical features were included in section A. The questions related to the factor of postoperative pain were included in Section B, and they consisted of 28 items in five categories, namely, patient's personal factors, sociodemographic factors, organizational (hospital-related) factors, communication factors, and postoperative factors. The questions of section B were based on Likert scale and were determined based on the literature review and input of experts. The patient's personal factors, such as belief in expressing pain, belief in opioids, fear of addiction, and side effects, were primarily taken from “Barrier Questionnaire II” (BQII), which has been used in previous study [11]. Other categories of factors were generated by reviewing the latest articles. The Content Validity Index (CVI) of the questionnaire, based on the experts' remarks, emerged as 0.91 for relevance and 0.87 for clarity. Cronbach's alpha was calculated and the reliability coefficient was measured as 0.785.

### 2.6. Data Collection and Management

The period of data collection was from April 2016 to June 2016. The score readings on pain were collected from the patients' charts for the 1st, 2nd, and 3rd POD, to assess the overall intensity of pain, and then participants were then asked to fill in the questionnaire. EpiData software version 3.1 was used for data entry, and questions with multiple answers were coded. Furthermore, double entry and data cleaning were also carried out by the expert.

### 2.7. Data Analysis

Data analysis was completed by using the statistical package for social sciences (SPSS) v.19 and STATA (Stata Corp LP, College Station, TX, USA) v.12. Descriptive statistics were computed for the demographic data, and factors of the pain and pain management. The frequency and percentage were computed for all the categorical variables, and mean (±SD) was computed for all the continuous variables. The pain scores of each POD were assessed for the normality using Kolmogorov-Smirnov (K-S) goodness of fit test. The mean (±SD) was also computed for three days' pain scores. The Mann-Whitney U test was applied to all the variables having two categories along with some of the demographic, clinical, and other potential variables. Kruskal Wallis test was computed for demographic and clinical variables having more than two categories. Furthermore, Spearman's rank correlation coefficient was applied for determining the association between all the continuous variables. The p-value of less than 0.05 was considered as significant throughout the analysis.

### 2.8. Ethical Considerations

Formal approval of the study was taken from the Ethical Review Committee (ERC) of the Aga Khan University. All participants were approached and selected on a voluntary basis and they signed the informed consent form prior to the filling of the questionnaire. All the participants of the study were provided with detailed information regarding the purpose, risks, benefits of the study, and right of withdrawal. The confidentiality of participants' information was secured and soft data was saved on computers with password protection.

## 3. Results

### 3.1. Demographic Characteristics

The sociodemographic features of the cardiac surgery patients are presented in Table 1. A total of 136 cardiac surgery patients aged 20-86 years participated in this research. The study showed that 82.4 % (n=112) of the participants were males. The mean (± SD) age of the subjects was 57.41(± 11.83), and 94.1% (n=128) were married. The ethnicities (languages) of the study participants are summarized in Table 1.

### 3.2. Clinical Characteristics of Study Participants

Some important clinical variables are shown in Table 2. The mean (± SD) BMI was 27.2 (± 4.86) and the majority of patients were falling in the category of either overweight or obese. The maximum number of patients, 82.4% (n=112), had CABG. Among all the patients, 26.5% (n=36) had a sedentary lifestyle, 29.4% (n=40) were doing mild exercise for one to three days, 29.4% (n=40) were doing moderate exercise for four to five days, and 14.5% (n=20) were very active and exercising daily.

### 3.3. Pain Scores

The pain levels of patients were noted from the bed-side flow sheets of the first, second, and third postoperative days (POD), which were assessed and recorded by the assigned nurses. The mean (±SD) pain levels on the first, second, and third PODs were 2.98 (±0.34), 2.96 (±0.36), and 2.98 (±0.48), respectively.

### 3.4. Inferential Statistics for Demographic Variables

The Mann-Whitney U, Kruskal Wallis, and Spearman test were applied to determine the relationship of pain scores with all the dependent variables, as shown in Table 3. The Spearman test was applied on the BMI, which showed a significant (0.036) association with the pain levels of third PODs. The Kruskal Wallis test was applied to the variable type of surgery which was also identified as significant (0.049) with the pain score of the third POD. The remaining demographic variables were found to be insignificant (not <0.05).

### 3.5. Factors Associated with Pain Scores

#### 3.5.1. Sociodemographic Factors

As shown in Table 4, 68.4% (n=93) of the patients perceived that frequent use of pain medication is considered bad in their culture. Similarly, 61.8% (n=84) believed that their age was not appropriate for the regular use of pain medication. Furthermore, 46.3% (n=63) stated that they avoided painkillers because of their high cost. The questions from the sociodemographic characteristics did not show statistical significance (not <0.05) with the pain scores.

#### 3.5.2. Patients' Personal Factors

The results identified that the patients' beliefs were the important concerns of patients which are shown in Table 4. The results revealed that 49.3% (n=67) participants believed that pain was a good sign after surgery and it should not be suppressed. It was also identified that 23.5% (n=32) patients perceived that pain was an obvious part of surgery which cannot be cured. Results show that 88.2% (n=120) of the patients believed that they would get addicted to opioids drug and found to be of statistical significance, with a p-value of 0.032 on the third POD. The patients were also concerned about the adverse effects of the pain medication as 72.8% (n=99) of the patients considered that pain medication would harm their immune system, which was also found significant (0.007) with the pain levels of the second POD; while, 77.9% (n=106) believed that their liver would be affected, and 76.5% (n=104) believed that their muscle tone and daily activity would be affected by medication use. Moreover, 47.8% (n=65) perceived that painkillers delayed wound healing, and 66.2% (n=90) thought that medications could induce sleep. Furthermore, 62.5% (n=85) considered that pain medication would be harmful to their sexual life. 55% (n=75) participants avoided painkillers because of their strong belief in home remedies, and 83% (n=113) participants believed that other coping strategies, such as deep breathing, meditation, and music therapy, worked better than pain medications.

#### 3.5.3. Postoperative Factors

Most of the patients (73.5%, n=100) perceived that physical activity would increase their pain and 81.6% (n=111) believed that restricting physical activity was a good strategy to manage pain. The relationship of pain and postoperative physical activity showed a significantly high association with the pain levels of the second POD, as evident by the p-value of 0.001. However, the question related to activity restriction to manage pain was found to be insignificant (0.641, 0.840, 0.960), with each POD pain score.

#### 3.5.4. Organizational Factors

Sixty-three percent (n=86), of the participants, responded that they got adequate information on postoperative pain management during preoperative teaching. A small number of patients, 22.8% (n=31), perceived that hospital shave inadequate standards and policies about pain management. Furthermore, only 17.6% (n=24) of patients expressed that the process for carrying out the physician's order to give pain medication was time consuming. Related to the knowledge and practice of nurses about postoperative pain management, 49.3% (n=67) of the patients believed that nurses had limited knowledge and 40.4% (n=55) perceived that nurses were reluctant to give pain medication because they feared adverse effects. However, 24.3% (n=33) thought that the nurses' attitude and perceptions made them nervous about taking pain medication. Furthermore, 43.4% (n=59) reported that they had to wait for the pain medication because nurses were busy in other tasks.

The results also showed that 21.3% (n=29) believed that nurses did not give priority to pain management during postoperative care. However, 88.2% (n=120) participants felt that the training of nurses needs to include more education on postoperative pain and management of pain. Nurses' reluctance to administer pain medication, due to fear of adverse effects, was significantly (0.027) associated with the pain scores of the second POD. Another factor, about the need for enhancing of nurses' education, was also statistically significant (0.037). However, most of the questions were found to be insignificant (not<0.05).

#### 3.5.5. Pain Expression and Communication Factors

The results also showed that the patients had misconceptions about the expression of pain. Almost 15.4% (n=21) expressed that it was difficult to tell the nurse about the pain. However, 89.7% (n=122) patients believed that frequent reporting of pain would give a negative impression of their personality, as shown in Table 4.

## 4. Discussion

The study identified that BMI and the type of surgery impose a significant impact on postoperative pain scores, and pain scores in obese and overweight patients were higher than those with normal BMI. Similar association between BMI and pain have been also identified in other studies and argued that the surgery of obese patients is complex which causes injury to large tissue areas and prolongs the retraction period during surgery [19, 20]. Pain scores were also related to the type of surgery in the current study. The majority of the patients in the present study had CABG with IMA graft. However, this study was unable to identify which subtype of surgery was significantly associated with pain. A previous study identified that patients having CABG with Left Internal Mammary Artery (LIMA) and valvular surgery were more likely to have higher levels of pain than those having CABG without LIMA [21, 22]. The IMA grafting causes trauma to the internal thoracic cage, which increases the intensity of chest pain. Furthermore, muscular injury, sternal retraction, and neurological exasperation from the chest-tube drainage during CABG also cause chest pain [14].

In addition, the fear of addiction and adverse effects of pain medication were found to be significant personal factors associated with high pain scores in the present study population. The element of fear of addiction was consistent with a previous study conducted which found that 78% of cardiac surgery patients believed that the use of opioid medication would certainly cause drug dependency [11]. On the contrary, previous study also identified that 51% of patients believed that the side effects of pain medications are not severe; however, in the present study, the fear of adverse effects was also significant [11]. This could be due to cultural differences between the two studies. The patients from Pakistani cultures have a strong belief that the use of pharmacological medicine can cause side effects; therefore, they prefer herbal medicines and home remedies. Similarly, another study found that the severity of opioid side effects was perceived as more than its degree of pain control. Patients considered that the side effects caused by pain medication are more severe than the pain itself; therefore, they preferred to bear the pain rather than suffer from its side effects [23]. This could be a contributing factor in inadequate medication therapy.

Likewise, postoperative physical activity was another factor that had a statistically significant association with pain scores. In the current study, majority (73.5 %) of patients believed that postoperative activities were painful. Similar to the current study, the patients from previous studies also considered postoperative physical activities as painful; specifically, the cardiac surgery patients experienced pain with coughing, positioning in bed, ambulation, and breathing exercise and endeavored to restrict their activity to manage pain, thereby increasing the risk of complication [4, 6, 24].

Furthermore, in the current study, 40% of patients perceived that nurses are reluctant to give pain medication because of fear of adverse effects. Other studies also suggest that the nurses' attitude towards pain medication administration is a barrier to adequate pain management [9, 10]. Similarly, cardiac surgery patients in the current study felt that nurses should be educated more on pain management. These findings are in line with other studies conducted in Ghana and East Asian region, which demonstrated that pain management is influenced by multiple factors, such as preservice education, chance for in-service training, preparedness to learn, lack of training programmes and insufficient in-service training opportunities about pain management for nurses, and gaps in knowledge related to pain management [25]. Likewise, knowledge about the impact of cultural, age-related factors on pain and also having insufficient knowledge about analgesics were also reported as factors influencing pain management practices in the clinical settings [6]. In the current study, there could be a possibility that nurses who were taking care of cardiac surgery patients did not have the opportunities for in-service training on pain management. Therefore, this component of knowledge, related to pain management, is very important for the nurses taking care of cardiac surgery patients.

### 4.1. Strengths of the Study

To the best of the researchers' knowledge, this is the first study on this topic carried out in Pakistan. The questionnaire used in this study was found relevant because it was developed by analyzing the available literature on all the associated factors of pain and its management. The population of this study comprised all types of cardiac surgery patients, regardless of any specific type; therefore, this research could be replicated in other parts of the country as well. The data was collected by the principal investigator to reduce the chance of biases.

### 4.2. Limitations of the Study

The current study has some limitations; for example, the pain scores could be measured directly from the patients, during different activities, and at different time intervals. The anticipated sample size of the study could not be achieved because of time limitations. This might have affected the internal validity and power of the study. The study was limited to one setting; hence, this study has limited generalizability.

### 4.3. Recommendations for the Study

#### 4.3.1. Recommendations to Improve Clinical Practice

1. The patients preparing for elective cardiac surgery should be encouraged to work on their weight reduction to minimize the risk of severe postoperative pain.
2. Patients should be given adequate awareness with regards to the effects and side effects of opioid medication, to reduce their fears, and to promote compliance with pain medication, during hospitalization and after discharge.
3. The preoperative education plan of a patient should include the significance of postoperative physical activities and the rehabilitation plan, in order to improve its effectiveness.
4. Pain management should be incorporated as a separate unit in the nursing curriculum.

#### 4.3.2. Recommendations for Further Researches

1. Similar studies should be carried out in the private and public hospitals of the country on a larger scale, to improve the generalizability and to promote a better understanding of the phenomenon.
2. Studies may be conducted to validate the precision of the tools employed in this research.
3. Case-control or prospective cohort designs should be employed to identify the causal relationship between the variables. Furthermore, qualitative studies could be conducted to explore the phenomenon of pain management among healthcare providers and cardiac surgery patients in more depth.

## 5. Conclusion

Overall, this study reported a mild postoperative pain score for the first, second, and third postoperative days, among the cardiac surgery patients. The study also highlighted some of the facilitating and hindering factors related to pain management. The findings suggest the need for enhancement in preoperative education of the patients, especially, giving importance to weight reduction, pain medication, and postoperative physical activities. Furthermore, the results also proposed the need for improvement in educating the nurses about postoperative pain management.

## Figures and Tables

**Table 1:** Demographic Characteristics of the Cardiac Surgery Patients (n=136).

Characteristics	N	(%)
*Age (in years)* Mean ± SD	57.41	**±**11.8

*Gender*		
Male	112	(82.4)
Female	24	(17.6)

*Occupation*		
Housewife	18	(13.2)
Personal Business	40	(29.4)
Government Job	11	(8.1)
Retired	30	(22.1)
Student	3	(2.2)
Private Job	34	(25.0)

*Family Income (PKR per month)*		
Less than 14,999	27	(19.9)
15,000- 29,999	33	(24.3)
30,000-44,999	13	(9.6)
45,000-59,999	15	(11.0)
60,000 or more	48	(35.3)

*Marital Status*		
Single	2	(1.5)
Married	128	(94.1)
Widow/Widower	6	(4.4)

*Academic Qualification*		
Primary	47	(34.6)
Secondary	15	(11.0)
Intermediate/ “A” Level	12	(8.8)
Bachelor	43	(32.4)
Master	18	(13.2)

*Ethnicity (Language)*		
Muhajir (Urdu Speaking)	35	(25.7)
Sindhi	37	(27.2)
Punjabi	20	(14.7)
Pathan	10	(7.4)
Balochi	10	(7.4)
Others^a^	24	(17.6)

^a^others include Afghani, Baroi, Chitrali, Gilgiti, Gujrati, Hazara, Kachi, Kashmiri, Memon, Pothwari, & Sariki.

**Table 2:** Clinical Characteristics of the Cardiac Surgery Patients (n=136).

Characteristics	N	(%)
*BMI* Mean ± SD	27.27	**±**4.86
Underweight (<18.5)	1	(0.7)
Healthy Weight (18.5-24.9)	48	(35.3)
Overweight (25-30)	51	(37.5)
Obese (>30)	35	(25.7)

*Presence of Co-morbidity *		
Yes	108	(79.4)
No	28	(20.6)

*Type of Surgery*		
CABG	112	(82.4)
Congenital Heart Surgeries	5	(3.7)
Valvular Heart Surgeries	15	(11.0)
CABG and Valvular Heart Surgeries	4	(2.9)

*Activity Level (before Surgery)*		
Sedentary (little to no exercise)	36	(26.5)
Light (1-3 days per week)	40	(29.4)
Moderate (4-5 days per week)	40	(29.4)
Very Active (6-7 days per week)	20	(14.7)

**Table 3:** Analysis of Demographic and Clinical Characteristics with Pain Scores (n=136).

Variables	1^st^ POD pain levels	2^nd^ POD pain levels	3^rd^ POD pain levels
	*P*-values		
^a^Gender	0.470	0.208	0.819
^c^Age	0.965	0.636	0.117
^c^BMI	0.214	0.796	0.036^*∗*^
^b^Occupation	0.571	0.337	0.902
^b^Income	0.991	0.185	0.591
^b^Marital Status	0.741	0.647	0.832
^b^Academic Qualification	0.615	0.540	0.219
^b^Ethnicity	0.418	0.348	0.948
^b^Type of Surgery	0.405	0.056	0.049^*∗*^
^b^Activity Levels (before surgery)	0.352	0.214	0.423
^a^Presence of Co-morbidity	0.244	0.700	0.462

^*∗*^ *P-*value < 0.05 ^a^Mann-Whitney test ^b^Kruskal Wallis test ^c^Spearman test

**Table 4:** Descriptive Statistics of factors of pain and its management after cardiac surgery (n=136).

	n (%)	
	Yes	No
Socio-demographic factors		

Repudiation of pain medication in culture	93(68.4)	43(31.6)
Age inappropriate to take pain medication frequently	84(61.8)	52(38.2)
Pain medications are very expensive	63(46.3)	73(53.7)

Personal Factors		

pain medications are inappropriate after cardiac surgery	25(18.4)	111(81.6)
Pain should not be suppressed Post-operatively	67(49.3)	69(50.7)
Post-operative pain is inevitable	32(23.5)	104(76.5)
Easy addiction with opioid analgesics	120(88.2)^*∗*^	16(11.8)
Pain medication affects the immune system	99(72.8)^*∗*^	37(27.2)
Pain medication affects liver	106(77.9)	30(22.1)
Pain medication affects muscle tone and daily activity	104(76.5)	32(23.5)
Pain medication delays my wound healing	65(47.8)	71(52.2)
Pain medication causes drowsiness	90(66.2)	46(33.8)
Pain medication decreases libido	85(62.5)	51(37.5)

Home remedies are better than pain medications	75(55.1)	61(44.9)
Deep breathing, meditation, and music therapy are better than pain medication	113(83.1)	23(16.9)

Post-operative factors		

Physical activity increases pain	100(73.5)^*∗*^	36(26.5)
Activity restriction is good to manage pain	111(81.6)	25(18.4)

Organizational factors		

Adequate pre-operative teaching about post-operative pain and its management	86(63.2)	50(36.8)
Inadequate policy and standards about post-operative pain and its management	31(22.8)	105(77.2)
Medication process is time consuming	24(17.6)	112(82.4)
Nurses' perception and attitude make patients nervous about taking painkillers	33(24.3)	103(75.7)
Nurses workload delays pain management	59(43.4)	77(56.6)
Nurses neglect pain and its management after cardiac surgeries	29(21.3)	107(78.7)
Nurses are reluctant to give painkillers	55(40.4)^*∗*^	81(59.6)
Nurses are ignorant about post-operative pain and its management	67(49.3)	69(50.7)
Nurses need need more education about pain and its management after cardiac surgery	120(88.2)^*∗*^	16(11.8)

Pain Expression and Communication Factors		

Feeling difficulty to tell a nurse about pain	21(15.4)	115(84.6)
Reporting pain repetitively gives an impact of an irritating personality	122(89.7)	14(10.3)

^*∗*^ *P-*value < 0.05

## Data Availability

The data used to support the findings of this study are available from the corresponding author upon request.
